# PS-35 - PREDICTORS OF DELAYED SECOND MMR DOSE UPTAKE AMONG CHILDREN TURNING FIVE BETWEEN 2023 AND 2025 IN SCOTLAND

**DOI:** 10.1093/pubmed/fdag046.072

**Published:** 2026-07-09

**Authors:** Dr Folakemi Abiodun Osundare, Dr Will Morton, Gareth J Hughes, Dr Taimoor Hasan, Dr Cheryl L Gibbons, Dr Sam Ghebrehewet

**Affiliations:** Public Health Scotland; UK Health Security Agency; UK Health Security Agency; Public Health Scotland; Public Health Scotland; Public Health Scotland

## Abstract

**Background:**

The World Health Organization recommends a second dose of measles-containing vaccine (MCV), regardless of primary MCV coverage. Timely administration of the second dose of the Measles, Mumps, Rubella vaccine (MMR2) at 3 years and 4 months of age is crucial in reducing susceptibility to measles virus, particularly among children who did not respond to, or did not receive, the first MMR dose at 12 months of age. While many socio-demographic and household factors associated with delays in childhood vaccination are known, the specific determinants of delayed MMR2 uptake are less well understood.

**Objectives:**

To identify determinants of delayed MMR2 vaccination among children turning five between 2023 and 2025 in Scotland.

**Method:**

This was a retrospective cohort study. Data from the Scottish Linked Pregnancy and Baby Dataset (SLiPBD), general/acute inpatient and day-case records (SMR01), the Child Health System, and vaccination events in the Scottish Immunisation Recall System (SIRS) were linked. Descriptive analyses compared timely versus delayed MMR2 uptake and regression models identified factors independently associated with delayed MMR2 uptake, defined as receipt later than 3 years 5 months.

**Result:**

Incomplete uptake of other primary childhood vaccinations was the strongest predictor of delayed MMR2 uptake (adjusted odds Ratio (aOR) 2.19; 95%CI: 1.99-2.40). Other factors were: younger maternal age (≤29 years: aORs 1.07-1.62), maternal smoking (aOR 1.41; 95%CI:1.33–1.49), multiparity (aOR 1.63; 95%CI:1.54-1.72), lower socioeconomic status (aORs 1.06–1.33), non-native English homes (aOR 1.17; 95% CI:(1.08–1.26), hospital visit around vaccination time (aOR 1.16; 95%CI:(1.11–1.24), and vaccination in spring (aOR 1.73; 95%CI 1.64-1.83).

**Conclusion:**

Delayed MMR2 uptake in Scotland is associated with incomplete primary vaccination, as well as maternal, socioeconomic, and health factors. These insights could support targeted interventions for under-vaccinated and vulnerable families to improve uptake, timeliness, reduce susceptibility, and support measles elimination goals.

